# Sex-specific differences in pancreatic α-cell function

**DOI:** 10.3389/fendo.2026.1936339

**Published:** 2026-08-26

**Authors:** Emmanuel Ampofo, Selina Wrublewsky, Matthias W. Laschke

**Affiliations:** 1 Institute for Clinical and Experimental Surgery, PharmaScienceHub (PSH), Saarland University, Homburg, Germany; 2 Center for Gender-specific Biology and Medicine (CGBM), Saarland University, Homburg, Germany

**Keywords:** glucagon, islets of langerhans, pancreas, sex, α-cells

## Abstract

Although sex is a critical biological variable determining physiology, disease risk and therapeutic responses, it remains underrepresented in biomedical research. While sex-dependent differences are well established in cardiovascular and metabolic diseases, their impact on glucagon biology is less understood. Glucagon is the key counter-regulatory hormone to insulin and plays a central role in glucose homeostasis by regulating hepatic glucose production. To date, only a limited number of studies have addressed sex-specific differences in glucagon plasma levels, secretion and α-cell function, indicating that females tend to exhibit a higher glucagon secretion. For the first time, this review summarizes the current literature on glucagon biology suggesting a sex-biased regulation of α-cell activity.

## Introduction

Sex is a fundamental biological variable that determines physiology, disease susceptibility and therapeutic responses. Nevertheless, for decades, biomedical research has largely relied on male-based models, assuming that its findings can be generalized to both sexes. This paradigm is increasingly challenged by evidence demonstrating pervasive sex-specific differences across organ systems, extending far beyond reproductive biology (1).

Cardiovascular diseases provide a striking example. While men develop coronary artery disease earlier, women often present with atypical symptoms and experience worse outcomes following myocardial infarction (2). This indicates the need for sex-tailored diagnostic and therapeutic strategies (2). Sex-dependent biology is evident even before birth. Male preterm infants exhibit higher morbidity and mortality when compared to females caused by an increased susceptibility to respiratory distress, impaired lung maturation and altered inflammatory responses (3, 4). This suggests that sex-specific developmental programs are established early and are not solely driven by gonadal hormones. In fact, many studies already reported that non-reproductive hormonal systems contribute substantially to sex-dependent physiology. Adipokines, such as leptin and adiponectin, display robust sex differences, independent of total fat mass (5). Females typically exhibit higher circulating levels of both hormones, consistent with a predominance of subcutaneous adipose tissue and a more favorable metabolic and inflammatory profile (6). In contrast, males accumulate more visceral fat, which is associated with chronic low-grade inflammation and metabolic dysfunction (7).

Such sex-dependent differences in adipose tissue biology and systemic inflammation are closely linked to alterations in glucose metabolism and insulin sensitivity. Numerous clinical and experimental findings indicate that women, particularly premenopausal women, tend to display stronger insulin sensitivity when compared to men (8–10). Conversely, men are more prone to developing insulin resistance and type 2 diabetes (T2DM), often at lower body mass indices (10). While insulin has been extensively studied in the context of sex-dependent metabolism (11–14), considerably less attention has been given to glucagon, the primary counter-regulatory hormone that opposes insulin action. Glucagon plays a central role in maintaining glucose homeostasis by promoting hepatic glucose production through glycogenolysis and gluconeogenesis (15, 16). Emerging evidence suggests that glucagon plasma levels, glucagon secretion and α-cell mass may differ between males and females, although the available data remain limited and sometimes contradictory. For the first time, the present review provides an overview of these potential sex-dependent differences in glucagon biology.

## Effect of sex on glucagon plasma levels

Glucagon is a 29-amino-acid peptide hormone secreted by pancreatic α-cells. It is generated from the proglucagon precursor through enzymatic cleavage by prohormone convertase 2, which also produces other peptides, such as glucagon like peptide (GLP)-1 and oxyntomodulin. Under physiological conditions, human plasma glucagon levels range from ~7.4-10 pmol/L (17), whereas in mice, they typically range from ~10-30 pmol/L (18). Glucagon is traditionally considered a counter-regulatory hormone to insulin, primarily acting in peripheral tissues, such as the liver, where it fine-tunes the balance between gluconeogenesis and glycogenolysis. In hepatocytes, glucagon signaling is highly complex, involving the coordinated modulation of transcriptional programs and intracellular signaling pathways that collectively regulate amino acid, lipid and carbohydrate metabolism.

To date, there are only a few studies reporting sex-specific differences in plasma glucagon levels. Karlsson et al. (19) found that plasma glucagon levels in response to 2-deoxy-d-glucose-induced neuroglycopenia are markedly higher in female when compared to male mice. Moreover, glucagon levels are higher in fasted female when compared to male mice (20). Horie et al. (12) analyzed glucagon levels in young healthy adults. They found higher glucagon levels in female adults when compared to male adults. The authors speculated that this is due to slower gut peristalsis (21), resulting in delayed glucose absorption in females (12). In contrast, Huang et al. (22) showed that males with T2DM display more pronounced hyperglucagonemia than females, particularly after a nutrient load. However, it should be noted that the differences in sex among individuals with T2DM also affect the progression of the disease (23). Taken together, these limited findings suggest that sex appears to influence glucagon levels with a tendency towards higher glucagon plasma levels in females.

## Effect of sex on glucagon secretion

Glucagon secretion is controlled by complex nutritional and neurohormonal factors. It is stimulated in response to hypoglycemia, various amino acids and glucose-dependent insulinotropic polypeptide (GIP) and inhibited by somatostatin, gamma-aminobutyric acid (GABA) and leptin. However, whether sex may affect glucagon secretion is not fully understood. Only a few studies reported that female sex hormones may negatively affect glucagon expression and secretion. For instance, estradiol decreases glucagon secretion in streptozotocin-treated male mice transplanted with human or mouse islets (24, 25). In this context, Handgraaf et al. (26) reported that estradiol directly acts on isolated α-cells through all estrogen receptors. This leads to a changed maturation of pro-glucagon-derived peptides to increase GLP-1 and decrease glucagon gene expression and secretion. However, these studies did not compare hormone-reduced glucagon secretion in female islets with that from male islets. On the other hand, Karlsson et al. (19) analyzed sex differences in the glucagon response in detail. They found an increased sensitivity of the glucagon-producing α-cells of isolated islets from female mice to cholinergic stimulation (19).

Recent single-cell transcriptomic and epigenomic studies provide additional insight into sex-dependent gene expression patterns within pancreatic islets. For instance, the group of Wang examined sex differences in the genetic program of pancreatic endocrine cells (27). They identified sex-associated transcriptional differences across the cell populations, indicating that sex influences islet function at the level of cell type-specific gene regulatory programs (27). More recently, Qadir et al. (28) combined single-cell RNA-sequencing with single-nucleus assay for transposase-accessible chromatin (ATAC)-sequencing in human islets from donors with and without T2DM. In non-diabetic donors, sex differences in gene expression and chromatin accessibility predominantly involved sex chromosome-associated genes, whereas additional sex-dependent alterations in autosomal genes emerged in T2DM. These findings indicate that sex-dependent differences in endocrine cell function are associated with distinct transcriptional and regulatory states and may become particularly pronounced under metabolic stress.

Our group demonstrated a significantly higher expression and secretion of glucagon in isolated murine female islets when compared to male islets (29). This, in turn, activated angiogenic pathways in female endothelial cells and pericytes promoting the development of an islet microvasculature (29). Recently, the group of Guy A. Rutter (30) investigated sex-specific α-cell Ca²^+^ dynamics. They demonstrated that female α-cells exhibit an enhanced sensitivity to somatostatin-mediated signaling, whereas male α-cells are more responsive to insulin receptor signaling. The authors therefore proposed that α-cell excitability is finely tuned by inhibitory signals arising from the close juxtaposition of β- and δ-cells (30), which may contribute to elevated glucagon secretion in female mice. Consistent with these findings, a positive effect of female sex on glucagon secretion and expression has also been observed in human islets. Isolated islets from women showed a tendency toward increased glucagon secretion in response to classical secretagogues (28). These observations suggest that female islets may be more sensitive to extracellular stimuli that trigger glucagon release.

Nagai et al. (31) reported sex differences in the expression of sodium-glucose cotransporters (SGLT1 and SGLT2) in several murine tissues, including higher renal expression in females. However, whether such sex-dependent expression also occurs specifically in pancreatic α-cells has not been demonstrated. Of note, SGLT1 is expressed in pancreatic α-cells and has been shown to contribute to the regulation of glucagon secretion (32). In contrast, the expression and function of SGLT2 in α-cells remain controversial. While Bonner et al. (33) reported SGLT2 expression in human α-cells and proposed that its pharmacological inhibition directly stimulates glucagon secretion, subsequent studies failed to detect significant SGLT2 expression in rodent and human α-cells and did not observe a direct effect of SGLT2 inhibition on glucagon secretion (34). Thus, current evidence does not support the assumption that sex-dependent differences in α-cell SGLT1 or SGLT2 expression contribute to the increased glucagon secretion observed in females and further cell-specific studies are required to address this possibility.

## Effect of sex on pancreatic α-cell mass

**Figure 1:**
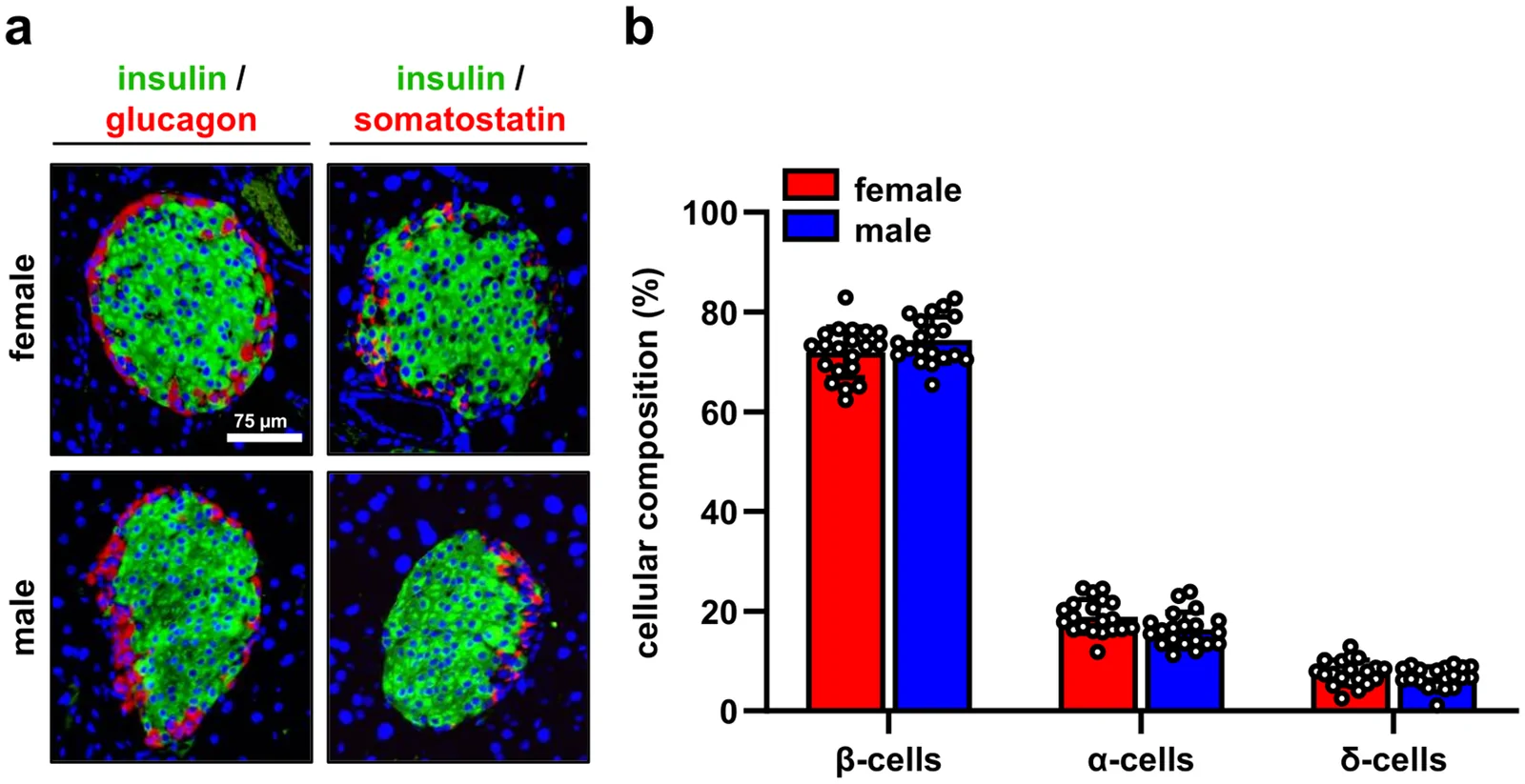
Cellular composition of islets within the pancreas. **(A)** Representative immunofluorescent stainings of insulin/glucagon and insulin/somatostatin in pancreatic islets of male and female C57BL/6J mice. Cell nuclei were stained with Hoechst 33342 (blue). Scale bar: 75 µm. **(B)** Insulin- (β-cells), glucagon- (α-cells) and somatostatin- (δ-cells) positive cells in pancreatic islets of male and female C57BL/6J mice. Data are given in % of all islet cells (n = 20 islets each). Mean ± SEM.

The mass of α-cells is increasingly recognized as a highly dynamic and adaptive component of the islet microenvironment rather than as a static entity. A growing body of evidence indicates that the α-cell mass undergoes significant remodeling under various physiological and pathological conditions, including diabetes mellitus, altered nutrient availability and hormonal perturbations (16, 35–37), suggesting that sex-specific alterations in glucagon secretion may arise not only from changes in α-cell activity but also from shifts in α-cell mass and islet architecture. In the early 80s, Robert C. McEvoy (38) analyzed in detail changes in the volumes of α-, β-, and δ-cells of rat pancreas from 10 days postnatal until 7 months of age. He reported that neither islet hormone concentrations nor the relative proportions of endocrine cell types differed between sexes at any age. Consistent with these observations, an equal distribution of α-, β-, and δ-cells is also observed in murine islets (**Figure 1**). Similarly, Dharmesh et al. (39) reported comparable numbers of α-cells in pancreatic islets of male and female goats. In contrast, other studies suggest sex-specific differences in the α-cell mass. In fact, several reports indicate that female mice exhibit an increased α-cell mass (19, 40). For instance, Crivello et al. (41) showed that female mice present with larger cluster sizes and an increased α-cell mass.

In humans, however, the evidence for sex-dependent differences in α-cell mass is less supportive. Rahier et al. (42) analyzed the endocrine pancreatic cell mass of 52 non-diabetic and 50 T2DM human subjects. As expected, the authors found a markedly decreased β-cell mass in T2DM subjects. However, the α-cell mass was not affected by sex. Similar results were found in a study investigating gene expression patterns by using next-generation RNA sequencing (43). Although adult islet composition varied among human donors, β-cell/α-cell ratios did not differ with age and sex (43).

Based on these findings, it cannot be excluded that biological sex also influences the α-cell mass. However, further studies are required to directly assess whether these differences reflect true alterations in α-cell mass or rather changes in cellular organization and functional adaptations within the islet microenvironment.

## Conclusions

**Figure 2:**
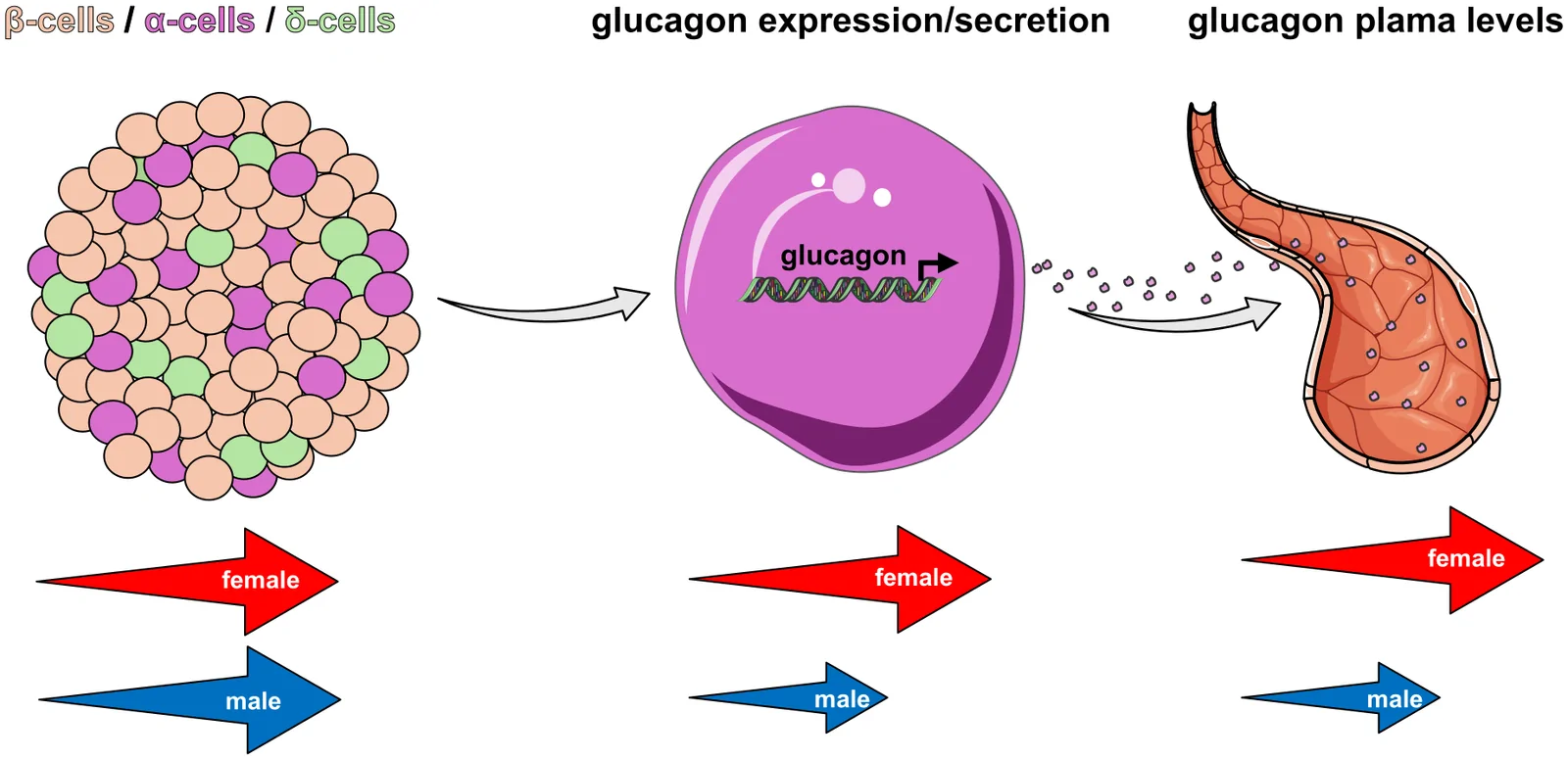
A proposed model of the glucagon biology in males and females. The current literature suggests that α-cell mass is not significantly influenced by sex. However, several studies indicate that glucagon expression and secretion, as well as plasma glucagon levels, are elevated in females.

The current literature suggests sex-dependent differences in glucagon biology. Several experimental and clinical studies indicates a tendency toward higher glucagon levels, expression and secretion in females (**Figure 2**). Recent single-cell transcriptomic and epigenomic studies further demonstrate sex-specific molecular programs within pancreatic endocrine cells. However, the direct role of sex hormones, such as androgens and progestogens, on α-cell activity must be further clarified in detail. Moreover, indirect effects by the interplay between glucagon signaling, adipokines and inflammatory pathways may represent a further critical yet underexplored axis in sex-specific metabolic control. Given the central role of glucagon in metabolic control and its dysregulation in diabetes, a deeper understanding of its sex-dependent regulation is urgently needed.

In clinical trials for diabetes therapy, male and female patients typically receive similar treatment regimens and sex-specific differences are rarely considered when evaluating therapeutic efficacy. In preclinical research, male mice are predominantly used to establish models of diabetes, as they are generally more susceptible to the disease than females. Consequently, sex should be systematically incorporated as a biological variable in both basic and clinical research, not only in studies of α-cell function, but across all aspects of diabetes research to ensure accurate assessment of therapeutic outcomes.
